# Isolation, Purification, and Identification of an Important Pigment, Sepiapterin, from Integument of the *lemon* Mutant of the Silkworm, *Bombyx mori*

**DOI:** 10.1673/031.013.11801

**Published:** 2013-11-27

**Authors:** Junshan Gao, Jing Wang, Wenjing Wang, Chaoliang Liu, Yan Meng

**Affiliations:** School of Life Sciences, Anhui Agricultural University, 130 West Changjiang Road, Hefei 230036, China

**Keywords:** BH4, biological synthesis, column chromatography, genetic resource, HPLC

## Abstract

Sepiapterin is the precursor of tetrahydrobiopterin, an important coenzyme of aromatic amino acid hydroxylases, the lack of which leads to a variety of physiological metabolic diseases or neurological syndromes in humans. Sepiapterin is a main pigment component in the integument of the *lemon* mutant of the silkworm, *Bombyx mori* (L.) (Lepidoptera: Bombycidae), and is present there in extremely high content, so *lemon* is a valuable genetic resource to extract sepiapterin. In this study, an effective experimental system was set up for isolation and purification of sepiapterin from *lemon* silkworms by optimizing homogenization solvent, elution buffer, and separation chromatographic column. The results showed that ethanol was the most suitable solvent to homogenize the integument, with a concentration of 50% and solid:liquid ratio of 1:20 (g/mL). Sepiapterin was purified successively by column chromatography of cellulose Ecteola, sephadex G-25-150, and cellulose phosphate, and was identified by ultraviolet-visible absorption spectrometry. A stable and accurate high performance liquid chromatography method was constructed to identify sepiapterin and conduct qualitative and quantitative analyses. Sepiapterin of high purity was achieved, and the harvest reached about 40 ug/g of integument in the experiments. This work helps to obtaining natural sepiapterin in large amounts in order to use the *lemon B. mori* mutant to produce BH4 *in vitro*.

## Introduction

Sepiapterin (SP) is a yellow pteridine pigment initially found in the *sepia* mutant of *Drosophila melanogaster* (Forrest et al. 1954). SP is one of the endogenous pigments that form an insect's body color and is an intermediate product in the metabolic pathway of tetrahydrobiopterin (BH4) (Thony et al. 2000). BH4 is an important coenzyme of aromatic amino acid hydroxylases and nitric-oxide synthase. BH4 is related to the evaluation of endothelial functions (Forgione et al. 2000; Maita et al. 2002). Reduction of the level and bioavailability of BH4 leads to endothelial dysfunction in such diseases as hypertension (Hong et al. 2001; Higashi et al. 2002; Zheng et al. 2003), glycuresis (Shinozaki et al. 2000; Guzik et al. 2002; Alp et al. 2003), and arteriosclerosis (Stroes et al. 1997). Moreover, disorder of synthesis and regeneration of BH4 could cause some physiological diseases, such as phenylketonuria and dopa-responsive dystonia (Kure et al. 1999; Trefz et al 2000). Research has suggested that BH4 deficiency-dependent diseases could be improved by oral supply of BH4 (Shinozaki et al. 2000; Higashi et al. 2002; Dhillon et al. 2003). As the precursor of BH4 in the salvage synthesis pathway, supplementation of SP could improve diseases that are due to the lack of BH4 (Pannirselvam et al. 2002). Research has demonstrated that SP and BH4 are effective drugs to treat some human diseases. Commercial SP and BH4 are mainly produced through the chemical synthesis approach and are expensive.

**Figure 1.**
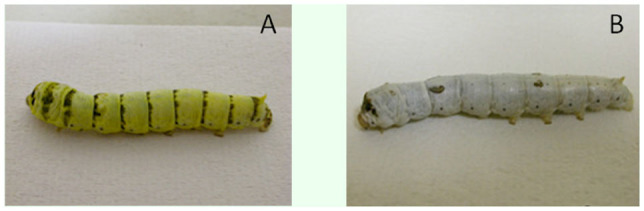
Larva of the *Bombyx mori lemon* mutant displays yellow body color (A), clearly different from that of normal silkworm strain (B). High quality figures are available online.

The *lemon* (*lem*) mutant is a body color mutant of the silkworm, *Bombyx mori* (L.) (Lepidoptera: Bombycidae), and displays yellow body coloration during larval developmental stages (Figure 1A), which is markedly different from body coloration of wild-type strains (Figure 1B). Previous studies showed that yellow pigments in the *lem* larvae contain SP, sepialumazine (deaminized product of SP), and riboflavin (Tsusue et al. 1965), of which SP is quantitatively major component accumulated in the integument (Tsujita et al.1955, 1961). Accumulation of SP in large amounts is due to a lack of the enzyme SP reductase in the mutant strain (Matsubara et al. 1963; Toshio et al. 1980). The loss of five amino acids at the carbon terminal of *B. mori* SP reductase is responsible for the *lem* phenotype (Meng et al. 2009).

The *lem* mutant is a valuable biological resource to extract SP. However, knowledge about extraction of SP from *lem* silkworms is limited. Methods used in previous studies are time-consuming and complex, and the efficiency of SP extraction and SP purity were not high (Tsusue et al. 1965; Gwen et al. 1979). In our study, an inexpensive, simple, optimized experimental system for the isolation and purification of SP from the integument of the *lem* mutant is introduced. A large number of silkworms can be reared on an artificial diet for many times in a year. This study makes sense for using biological synthesis instead of chemical synthesis of SP to produce BH4 *in vitro* at a large scale.

## Materials and Methods

### Silkworm strains, tissue and chemicals

The normal strain of p50 (Dazao) was maintained in the laboratory. The *lem* mutant strain of l70 was donated from Kyushu University (SilkwormBase, www.shigen.nig.ac.jp/silkwormbase/index.jsp). All larvae were fed fresh mulberry leaves under normal conditions (12:12 L:D, 25° C). On the fourth day of the 5^th^ instar, *lem* larvae were dissected in PBS in ice and the integument was separated from other tissues as clearly as possible and stored at -20° C for later use. SP standard (purity > 99.9%) and column filling materials of cellulose Ecteola, cellulose DEAE, cellulose AE, Sephadex G-25–150, and cellulose phosphate were obtained from Sigma-Aldrich (www.sigmaaldrich.com). Methanol and acetic acid used in the high performance liquid chromatography experiment were chromatographically pure (Sigma). All other reagents were of analytical grade.

**Figure 2.**
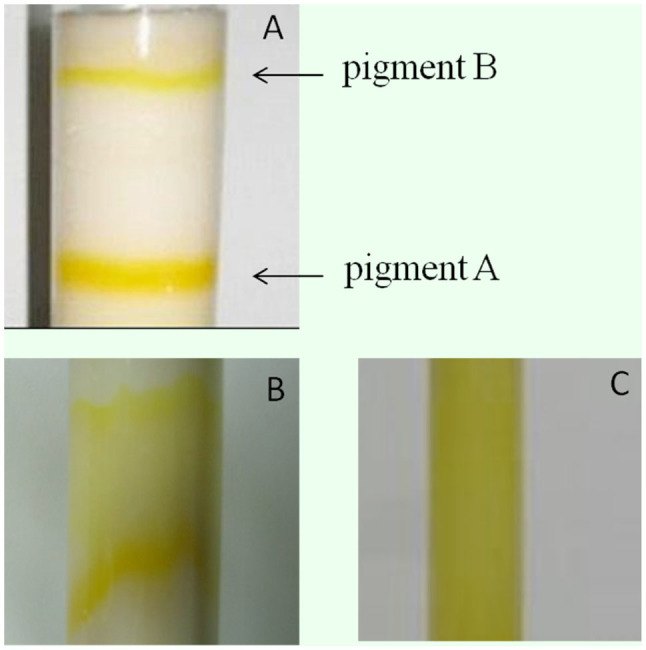
Comparison of the pigment separating effect among cellulose Ecteola (A, elution time, 7 hr), cellulose DEAE (B, 24 hr), and cellulose AE (C, 24 hr) columns eluated by deionized water. High quality figures are available online.

**Figure 3.**
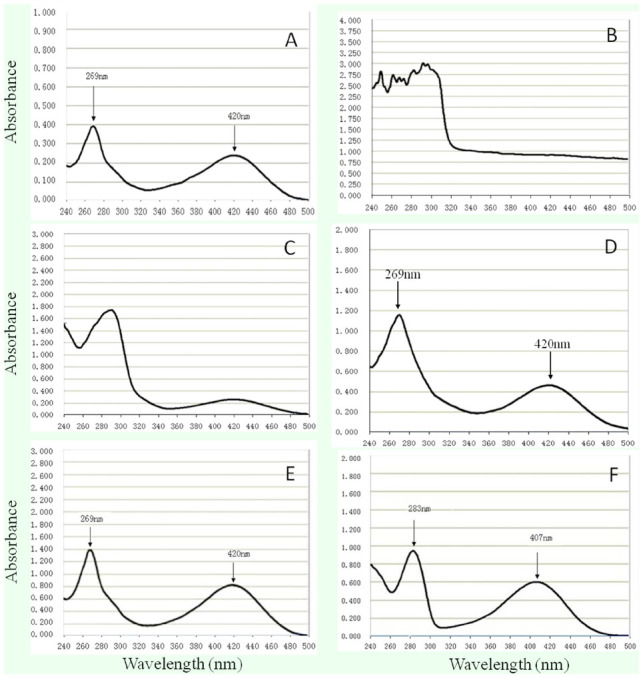
UV-Vis absorption spectra of standard SP (A), extracted mixture from *lem Bombyx mori* (B), pigment A after it was separated by cellulose Ecteola column (C), pigment A after it was purified by Sephadex G-25-150 column (D), pigment A after it was purified by cellulose phosphate column (E), and pigment B after it was purified by cellulose phosphate column (F). High quality figures are available online.

Common procedure for SP extraction 10 g of integument was used in every treatment. The tissue was homogenized by a Waring blender (www.waringproducts.com) with a certain volume of solvent for 10 min. After boiling for 10 min in a water bath with a reflux condenser, the homogenate was centrifuged at 15000 rpm for 30 min at 4° C . The supernatant was concentrated to 20 mL by a rotary evaporator (RE52-99, Yarong Biochemistry Instrument, www.shyarong.com), which was combined with a water cycle vacuum pump (SHZ-D, Yu Hua Instrument, www.gyyuhua.com). Then, the concentrate was conducted to separation column and purification column successively. Concentrate in each purification step was identified by an ultramicro spectrophotometer (NanoDrop 2000, Thermo Scientific, www.thermoscientific.com) at the wavelength of 420 nm, which is one of the maximum absorption peaks of SP (Figure 3A), to investigate the effect and quantity of SP extraction.

### Optimization of SP isolation system

To determine the most suitable lixiviating solvent, four kinds of liquids, deionized water (DW), 50% methanol, 50% ethanol, and 50% isopropylalcohol, were used in the experiment at solid:liquid ratio of 1:20 (g/mL). The best concentration of ethanol was decided by homogenizing the tissue with 30, 40, 50, 60, and 70% ethanol respectively at a solid:liquid ratio of 1:20. To optimize the relatively effective homogenization solid:liquid ratio, 50, 100, 150, 200, 250, 300, and 350 mL of 50% ethanol were used, respectively (i.e., the ratios from 1:10 to 1:35 (g/mL)) to treat the integument. To compare separating effects of SP from other pigments, three kinds of column filling ion exchange materials of cellulose Ecteola, cellulose DEAE, and cellulose AE were used as immobile phases. The size of the column was 2.8 × 28.5 cm. After all the pigments were put into column, DW was added to wash the column. In addition to continuous DW washing, elution with 0.01 M of acetic acid was performed after the pigments were separated completely by DW washing to compare the washing speed of the two methods.

### Purification and ultraviolet-visable identification of SP

As described previously with some modifications (Gwen et al. 1979), the fraction containing SP or sepialumazine obtained through cellulose Ecteola column separation was concentrated by the rotary evaporator, set to be dry powder, and redissolved in 10 mL DW. The resulting yellow solution was applied to a column of Sephadex G-25-150 (2.0 × 30.5 cm) and sequentially eluted with DW. The pigment-containing fraction was collected and concentrated again to be dry powder, redissolved in 5 mL DW, and applied to a column of cellulose phosphate (1.8 × 32.0 cm). The DW eluate was finally purified as a yellow pigment. Ultraviolet-visable (UV-Vis) absorption spectrum was carried out to check the purity of each eluate during the whole separation and purification procedure. According to the standard curve made by a commercial SP, the concentration of purified SP from *lem* silkworms was calculated.

### High performance liquid chromatography Assay

To perform qualitative and quantitative analysis of SP, a high performance liquid chromatography (HPLC) (LC-20A, Shimadzu, www.shimadzu.com) experimental system, in which ODSC_18_ (250 × 4.6 mm, 5um) was used as the chromatographic column (immobile phase), was optimized. The mobile phase was methanol-0.006% acetic acid (20:80, v/v) solution, with the flow rate of 1 mL per min to equilibrate the column. The column temperature was set at 25° C, and the detecting wavelength was 420 nm. 0.5 mM of standard SP flowed through the column six times to check the accuracy of the HPLC system. To confirm the stability of both the system and SP, 0.3 mM of standard SP was analyzed at 2, 4, 6, and 8 hr after being prepared. The retention time of the extracted pigments was compared for qualitative analysis under the same chromatographic condition. To calculate the quantity of extracted SP, a standard curve was plotted according to the peak areas of the standard SP in different concentrations.

## Results

### Optimization of the SP isolation system

SP had two absorption peaks at the wavelengths of 269 nm and 420 nm (Figure 3A). To optimize the SP isolation system, the isolating effects of different homogenization solvents of ethanol, methanol, isopropanol, and DW were investigated by measuring absorbance value of the concentrate at 420 nm. The result showed that using ethanol obtained the best effect among the four different solvents (data not shown). Further examinations indicated that 50% ethanol at a solid:liquid ratio of 1:20 (g/mL) produced comparatively better harvests according to absorbance value of the concentrate at 420 nm (data not shown). To obtain a more effective separation column, three kinds of cellulose of Ecteola, DEAE, and phosphate were screened as immobile phases. The results showed that cellulose Ecteola could separate the pigments into two clear regions, pigment A and pigment B (Figure 2A), and was more time-saving than cellulose DEAE (Figure 2B), while cellulose AE could not separate the pigments from each other at all (Figure 2C) by DW elution. Therefore, cellulose Ecteola was chosen in the following experiments. In addition, the speed of elution between DW and acetic acid was compared. As shown in Table 1, eluting the column with 0.01 M acetic acid after the pigments were separated completely with DW was more rapid, and the volume of eluent was markedly reduced, which could be easily concentrated compared to when DW alone was used.

### Purification and UV-Vis identification of SP

It seemed that the quantity of pigment A was higher than that of pigment B (Figure 2A), both of which were collected and purified by using the columns of Sephadex G-25-150 and cellulose phosphate, respectively. Figures 3C–E are the UV-Vis absorption spectrums of pigment A. It was apparent that the majority of other substances in the extraction mixture were removed by using the cellulose Ecteola column because miscellaneous peaks (Figure 3B) were mostly eliminated and the spectra of pigment A became regular with two main absorption peaks (Figure 3C), although it was still different from that of standard SP (Figure 3A). After purification by Sephadex G-25-150 column, pigment A displayed two maximum absorption peaks at about 269 nm and 420 nm (Figure 3D), which are the same as those of standard SP (Figure 3A), indicating that it had already been greatly purified. However, the lowest position between the two peaks (Figure 3D) was located at 340–360 nm, which was a slight shift when compared to that of standard SP (320–340 nm, Figure 3A), suggesting that there were still some impurities contained in pigment A. Therefore, a cellulose phosphate column was used to perform further purification. As a result, the UV-Vis absorption spectrum of pigment A (Figure 3E) was completely identical to that of standard SP (Figure 3A). The results demonstrated that the pigment A isolated by cellulose Ecteola column was in essence SP, while pigment B was probably a deaminized product of SP sepialumazine, because it had two other absorption peaks at 283 nm and 407 nm or so (Figure 3F). In addition, the volume of pigment A in each purification step was recorded, and the absorption value at 420 nm was determined (Table 2). According to the standard curve made using a commercial SP, concentration and quantity of purified SP from *lem* silkworms was calculated. SP was obtained at approximately 47 μg/g of integument of *lem* silkworms (Table 2).

**Table 1.**
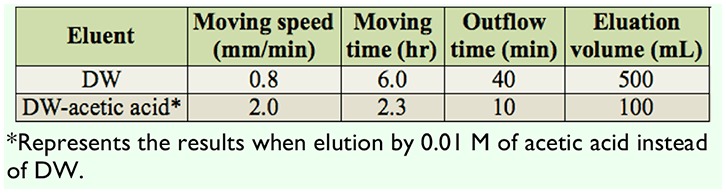
Comparison of elution effects of two mobile phases on pigment A.

**Table 2.**
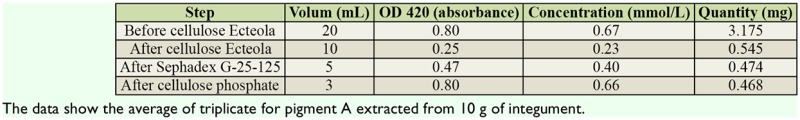
The quantity of pigment A in each purification step.

### HPLC analysis of SP

**Figure 4.**
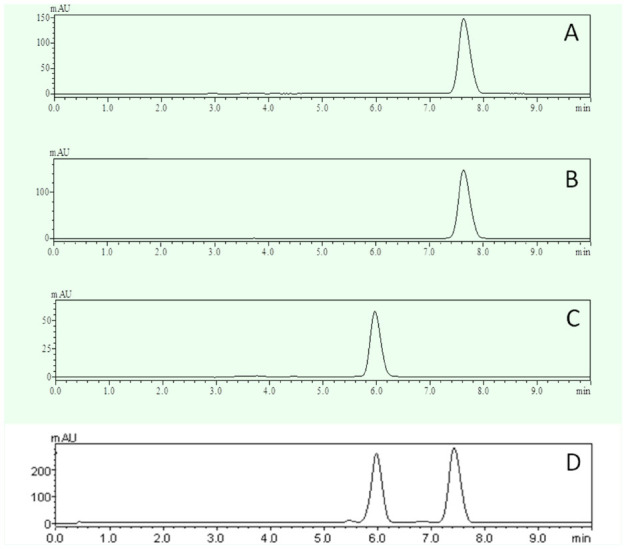
Retention time in the HPLC system for standard SP (A), pigment A (B), pigment B (C), and a mixture of pigments A and B (D). High quality figures are available online.

To further clarify that pigment A was SP and for a quantitative analysis of extracted SP, an HPLC experimental system was created, in which the mobile phase methanol-0.006% acetic acid was adjusted to the ratio of 20:80 (v/v) at the flow rate of 1 mL per min. To check the accuracy and stability of the system, standard SP at known concentrations was run through the HPLC column repeatedly at different time points (Table 3). It was clear that the peak areas were very similar to each other, indicating that the HPLC system was accurate and stable (Table 3). Next, the extracted pigments from *lem* silkworms were passed through the HPLC column to record the retention time. The results showed that the retenretention time of pigment A was ∼ 8 min (Figure 4B), which is consistent with that of standard SP (Figure 4A), while retention time of pigment B was ∼ 6 min (Figure 4C). The mixture of pigment A and B exhibited separate retention times of ∼ 8 and ∼ 6 min (Figure 4D). These data show that SP of high purity was successfully extracted from the silkworm mutant *lem*. The yield was about 38 ug/g of integument, according to the peak area of the SP sample (Table 4).

## Discussion

SP is a major pigment component in the body coloration of *lem*, a mutant of the silkworm, *B. mori* (Tsujita et al. 1961; Tsusue et al. 1965) (Figure 1A). The integument of *lem* larvae contains high concentrations of SP, so *lem* silkworms are an important resource to isolate SP as a natural bio-factory. However, methods for the extraction of SP are limited. In this study, an effective experimental system to isolate and purify SP from *lem* silkworm integument was established.

**Table 3.**
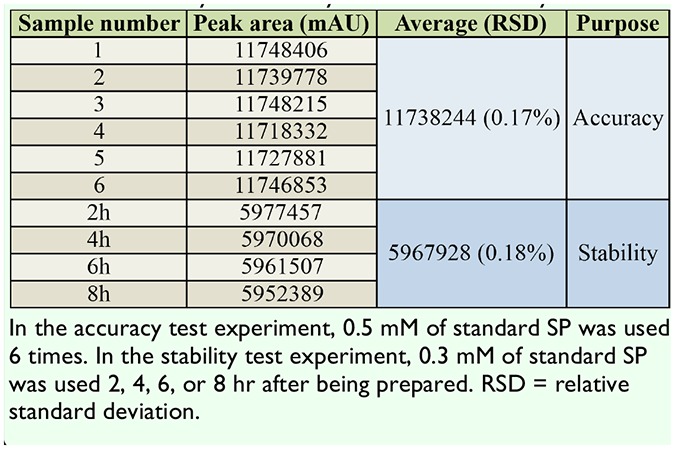
Accuracy and stability test on the HPLC system.

**Table 4.**
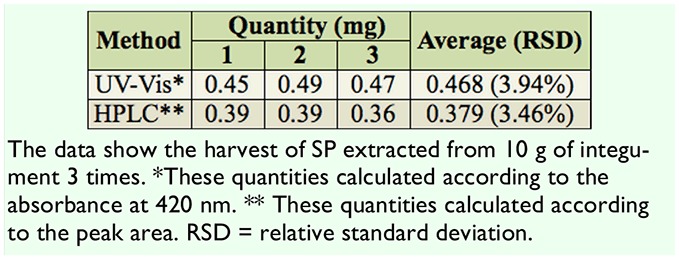
The quantity of purified SP in two methods.

In our study, two improvements were made on previous methods used for SP extraction (Tsusue et al. 1965, Gwen et al. 1979): 1) SP extraction procedures were systematically optimized among different homogenization solvents, solid:liquid ratios, and immobile phases, and 2) experimental steps were simpli fied, such as eliminating the treatments using chloroform and dilute ammonium hydroxide before applying pigment solution to the Ecteola cellulose column to increase the efficiency of SP extraction. By optimizing the isolation procedure of SP, 50% ethanol, which is cheap in price, non-toxic, and easily volatilized from the pigment (data not shown), was selected to homogenize the tissue of the integument at the solid:liquid ratio of 1:20 (g/L). Eluting the column with 0.01 M acetic acid after the pigments were separated completely with DW was a good gradient elution strategy because it shortened the separation time and decreased the elution volume (Table 1). As a negative ion-exchanger, cellulose Ecteola could not only separate SP from other pigments, but also eliminated the majority of impurities, including proteins and fat, playing an important role in SP purification (Figure 2A, 3B, C). The molecular sieve Sephadex G-25-150 and the cation exchanger cellulose phosphate had a relatively strong capability to adsorb macromolecules so that extracted SP was further purified to a perfect extent (Figure 3D, E). Moreover, the three column packing materials are all recyclable, which reduced the experimental cost. Using such a system, the harvest of SP reached to about 47 ug/g of integument of the *lem* silkworms (Table 4), which is a level that could bring considerable economic benefits.

By adjusting the mobile phase of methanol- 0.006% acetic acid to a more suitable ratio, a stable, accurate, and highly sensitive HPLC system was constructed for separating SP from other pigments and for SP identification according to the different retention times of each pigment component (Table 3, Figure 4). The SP harvest calculated by the peak area in HPLC was about 38 ug/g integument, slightly lower than that identified by OD 420, which could be due to normal loss during experiments (Table 4). Compared to the operations in traditional column chromatography, the HPLC method is simple, efficient, precise, and easily performed using a high-throughput HPLC system.

SP is the precursor of BH4, an important cofactor for aromatic amino acid hydroxylases and nitric-oxide synthase. Oral administration of SP and/or BH4 might improve some BH4 deficiency-dependent diseases (Shinozaki et al. 2000; Higashi et al. 2002; Pannirselvam et al. 2002; Dhillon et al. 2003). However, commercial SP and BH4 are mainly produced by chemical synthesis pathways that are expensive. It is necessary to set up an effective and cost-saving biopharmaceutical pathway to prepare SP and BH4 to replace the chemical synthesis pathway. The experimental system for SP isolation, purification, and identification from the silkworm *B. mori* that was established in this work is lower in cost, takes less time, produces a higher output, and may be medically safer than the chemical synthesis method. Aiming at utilizing novel *B. mori* genetic resources, the findings provide a scientific basis for industrially extracting natural SP from *lem* silkworms at a large scale.

## Abbreviations

**BH4,**: tetrahydrobiopterin;
**DW,**: deionized water;
**HPLC,**: high performance liquid chromatography;
***lem,***: *lemon;*
**SP,**: sepiapterin
